# Inhalation exposure-induced toxicity and disease mediated via mTOR dysregulation

**DOI:** 10.3389/ebm.2024.10135

**Published:** 2024-04-22

**Authors:** Akshada Shinde, Jonathan Shannahan

**Affiliations:** School of Health Sciences, College of Health and Human Sciences, Purdue University, West Lafayette, IN, United States

**Keywords:** mTOR, inhalation toxicology, lung disease, autophagy, lipid homeostasis, metabolism, inflammation

## Abstract

Environmental air pollution is a global health concern, associated with multiple respiratory and systemic diseases. Epidemiological supports continued urbanization and industrialization increasing the prevalence of inhalation exposures. Exposure to these inhaled pollutants induces toxicity via activation of numerous cellular mechanisms including oxidative stress, autophagy, disrupted cellular metabolism, inflammation, tumorigenesis, and others contributing to disease development. The mechanistic target of rapamycin (mTOR) is a key regulator involved in various cellular processes related to the modulation of metabolism and maintenance of homeostasis. Dysregulation of mTOR occurs following inhalation exposures and has also been implicated in many diseases such as cancer, obesity, cardiovascular disease, diabetes, asthma, and neurodegeneration. Moreover, mTOR plays a fundamental role in protein transcription and translation involved in many inflammatory and autoimmune diseases. It is necessary to understand inhalation exposure-induced dysregulation of mTOR since it is key regulator which may contribute to numerous disease processes. This mini review evaluates the available literature regarding several types of inhalation exposure and their impacts on mTOR signaling. Particularly we focus on the mTOR signaling pathway related outcomes of autophagy, lipid metabolism, and inflammation. Furthermore, we will examine the implications of dysregulated mTOR pathway in exposure-induced diseases. Throughout this mini review, current gaps will be identified related to exposure-induced mTOR dysregulation which may enable the targeting of mTOR signaling for the development of therapeutics.

## Impact statement

mTOR is a key regulator of numerous cellular processes often disrupted in numerous disease scenarios. Inhalation exposures are prevalent in environmental and occupational settings and contribute to both pulmonary and systemic diseases. Inhalation exposure-induced toxicity and diseases are associated with outcomes of mTOR dysregulation including autophagy, lipid metabolism, and inflammation. This minireview uniquely summarizes and integrates our current knowledge associated with inhalation exposure-indued dysregulation of mTOR contributing to toxicity and disease. This perspective is impactful as it highlights a complex and primary mediator to numerous disease processes contributing to human health risks associated with inhalation exposures. Further, a thorough understanding of mTOR-mediated mechanisms of toxicity and disease are necessary for the development of therapeutic strategies. Due to mTOR’s numerous cellular regulatory functions these strategies could be applied broadly to multiple exposures and disease scenarios.

## Introduction

Inhalation exposures are a significant global health concern associated with a variety of pulmonary and systemic diseases. Diseases associated with inhalation exposures include asthma, chronic obstructive pulmonary disease (COPD), pulmonary fibrosis, cancer, cardiovascular disease, and others [1]. Inhalation of environmental air pollution is associated with the induction of cellular responses including, oxidative stress, altered metabolism, autophagy, cell death and others. These cellular toxicity mechanisms contribute to disease development and progression [2]. For example, tobacco smoke exposure can result in development of COPD which is a complex lung disease involving destruction of the lung epithelium, bronchial inflammation, and fibrinogenesis. Individuals with COPD have demonstrated increased expression of microtubule-associated protein 1A/1B-light chain 3 (LC3) protein, a marker of autophagosome formation in lungs of COPD patients, suggesting of induction of autophagy [3]. Elucidation of molecular signaling mediating toxicity following inhalation exposures is necessary to develop interventional strategies to address health outcomes [4]. Mechanistic target of rapamycin (mTOR) represents a key regulator of multiple cellular processes, many of which are dysregulated following inhalation exposures which may contribute to the progression of disease. Specifically, mTOR regulates cell proliferation and survival, metabolism, autophagy, inflammation, oxidative stress, and DNA damage repair. Dysregulation of mTOR has been associated with numerous diseases including cancer, fibrosis, COPD, cardiovascular disease, autoimmune diseases, and metabolic disease [5, 6]. In this mini review we will assess the current literature regarding mTOR dysregulation associated with inhalation exposures and its contribution to toxicity and disease development. Additionally, we will highlight current knowledge gaps as well as the potential to target mTOR-mediated mechanisms for therapeutic strategies.

## mTOR complexes

**FIGURE 1:**
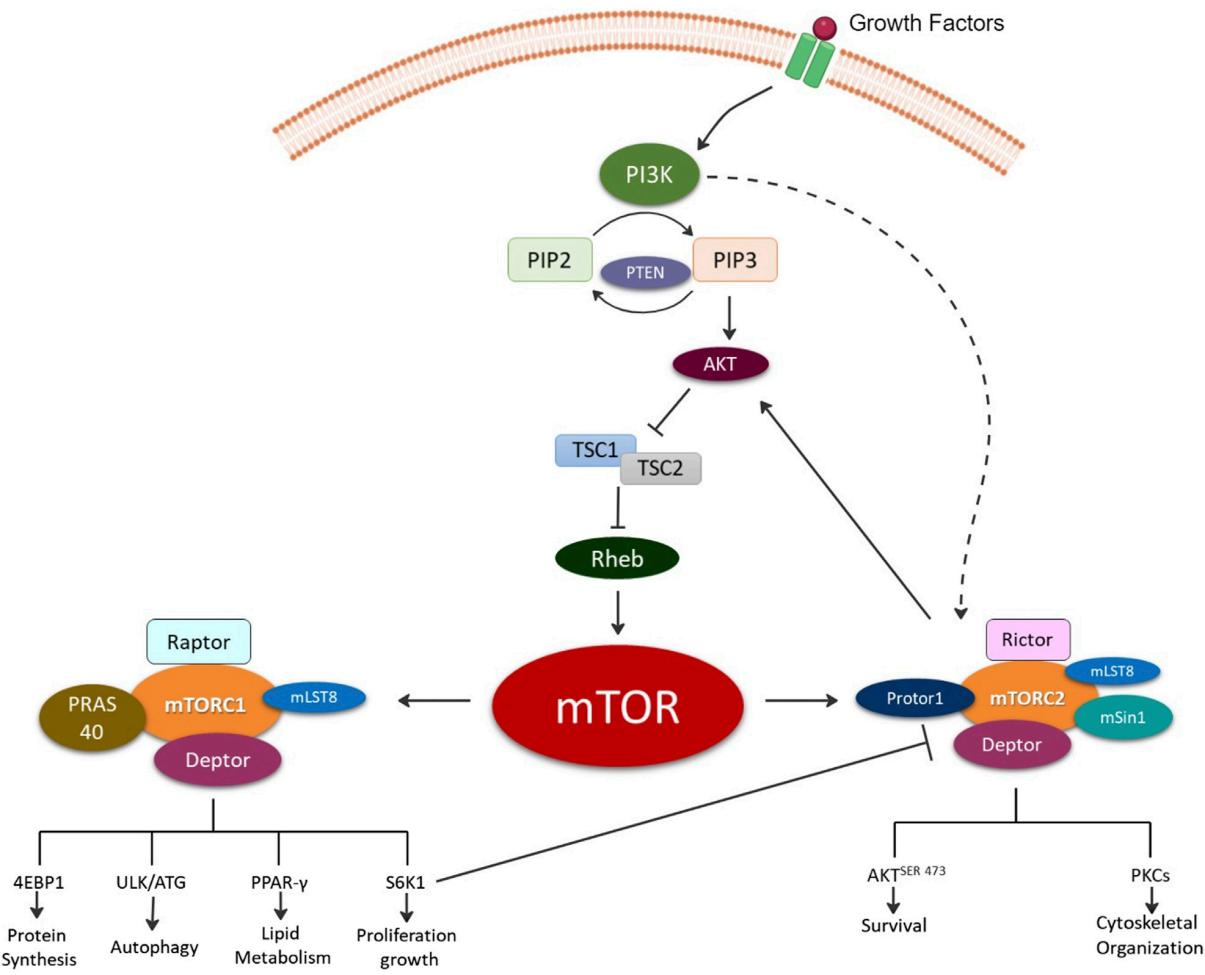
Illustration depicting the formation of the two mechanistic target of rapamycin (mTOR) complexes, mTOR complex 1 (mTORC1) and mTOR complex 2 (mTORC2). Briefly, growth factors activate, phosphoinositide 3-kinase (PI3K) signaling via production of phosphatidylinositol (3,4,5)-trisphosphate (PIP3) from phosphatidylinositol bisphosphate (PIP2) and phosphatase and tensin homolog (PTEN). PIP3 directly activates mTORC2 and indirectly mTORC1 via Akt (protein kinase B) activation and inhibition of tuberous sclerosis ½ (TSC ½) followed by Ras homolog enriched in brain (Rheb). The mTORC1 complex is composed of the rapamycin-sensitive adapter protein of mTOR (Raptor); DEP domain-containing mTOR-interacting protein (Deptor); proline-rich AKT substrate of 40KD (PRAS40); mTOR associated protein, and LST8 homolog (mLST8). mTORC1 signals for protein synthesis via Eukaryotic translation initiation factor 4E-binding protein 1 (4EBP1); autophagy via UNC51-like kinase 1 (ULK1); lipid metabolism regulation via Peroxisome proliferator-activated receptor gamma (PPAR-γ); and growth via ribosomal protein S6 kinase beta-1 (S6K1). mTORC2 complex is composed of the rapamycin-insensitive companion of mTOR (Rictor); Deptor; mLST8; proline-rich protein 5 (Protor-1); and stress-activated map kinase interacting protein 1 (mSin1). mTORC2 signals for cell survival via phosphorylation of protein kinase B at serine 473 (AKT^SER473^) and cytoskeletal organization via protein kinase C (PKC).

The discovery of rapamycin, a secondary metabolite originally observed to be produced by bacterium, led to a search for its cellular target, known as target of rapamycin (TOR) [7]. Several independent studies in 1994 led to the discovery the mammalian homologue of TOR or the mTOR [8]. mTOR is a 289-kDa serine/threonine kinase belonging to the phosphoinositide 3-kinases (PI3K)-related kinase family which is highly conserved across species. mTOR mediates cellular growth and proliferation via governing of intracellular adenosine triphosphate (ATP) levels based on extracellular nutrients. Further, it is involved in various cellular processes such as cell growth, survival, autophagy, and metabolism. These functions are mediated by two complexes mTOR complex 1 (mTORC1) and mTOR complex 2 (mTORC2) [5]. The upstream signaling resulting in formation of mTORC1 and mTORC2 as well as their distinct downstream signaling regulating cellular processes have previously been thoroughly reviewed elsewhere and are summarized in Figure 1 [8–14].

mTORC1 consists of the core subunits of 1) mTOR, 2) regulatory-associated protein of mTOR (Raptor), and 3) mammalian lethal with SEC13 protein 8 (mLST8) [15] (Figure 1). mTORC1 regulates biosynthesis of protein, lipids, and nucleic acids via transcriptional, translational, and post-translational mechanisms with the involvement of its substrate ribosomal protein S6 kinase (rpS6K) and eukaryotic translation initiation factor (elF) 4E–binding protein (4E-BP) [15]. It is also involved in ATP production via reducing NADPH and certain macromolecule precursors involved in biosynthesis [15]. Rapamycin and its related compounds, called rapalogs, are recognized as allosteric inhibitors of mTORC1. These include two endogenous inhibitors, DEP domain-containing mTOR-interacting protein (DEPTOR) and 40kDa Proline-rich Akt substrate (PRAS40). Rapamycin and other rapalogs primarily target mTORC1 making them useful in specifically regulating mTORC1 and its associated signaling enabling mechanistic investigations of mTORC1 signaling in laboratory settings.

mTORC2 consists of mTOR, DEPTOR, and mLST8, along with unique components Rapamycin-insensitive companion of mTOR (Rictor), protein-binding Rictor (Protor) and stress-activated protein kinase-interacting protein 1 (Sin1). Rictor’s primary function is the recruitment of substrates. mTORC2 does not bind Rapamycin nor impact S6K1. Thus, Raptor (mTORC1 subunit) and Rictor (mTORC2 subunit) independently associate with mTOR resulting in differential signaling outcomes and are utilized to define the complexes.

## Inhalation exposure modified mTOR signaling contributing to disease via autophagy

**FIGURE 2:**
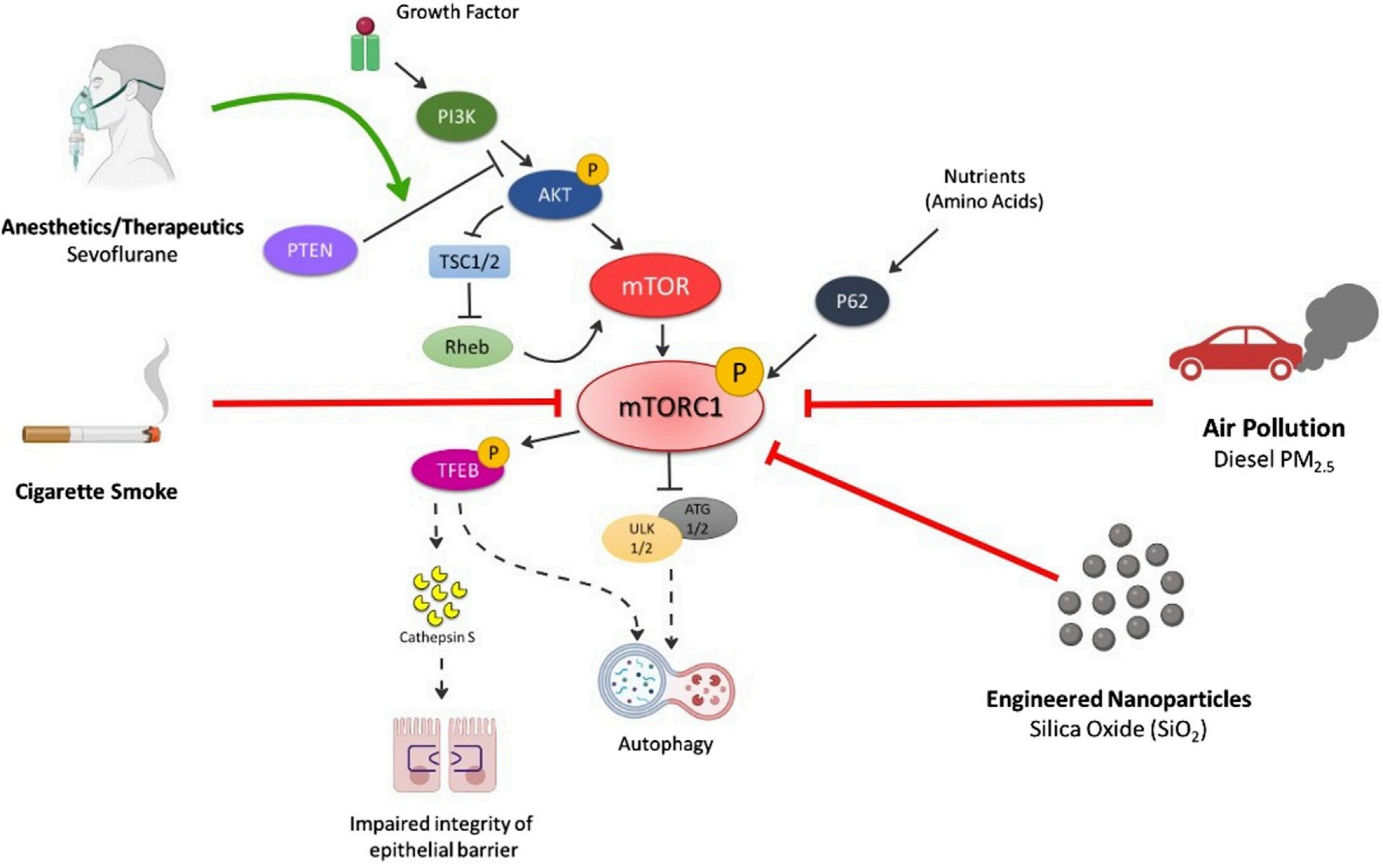
Summary of Inhalation-induced mechanisms by which various exposures cause dysregulation of mTORC1 and respective outcomes of cell death. Air pollution (especially PM_2.5_) inhibits p-PI3K, p-AKT, p-mTOR levels thereby activating autophagic cell death mechanism. Engineered nanoparticles (e.g., SiO_2_) decrease p-mTOR and p62 levels leading to autophagy. Cigarette smoke also reduces the levels of p-mTOR, decreasing p-TEFB, causing secretion of cathepsin S that impairs the integrity of lung epithelium and induction of inflammatory lung diseases. p-TEFB also acts as central regulator of autophagy and activates the pathway on mTOR dysregulation. Anesthetics (e.g., sevoflurane) activates autophagy via increase in PTEN/AKT/PI3K pathway. Solid black arrows—downstream signaling; Solid lines with end—inhibitory signal; Dotted black arrows—activates the pathway on mTOR inhibition/decrease; Solid Red line with end—Decrease in levels/inhibition of p-mTOR by exposures; Solid Green arrow—Activation of pathway.

Several inhalation exposures target the mTOR pathway, disrupting normal cellular and molecular pathways [6] (Figure 2). These exposures include commonly inhaled exposures including ambient particulate matter, engineered nanoparticles (NPs), anesthetics and therapeutics, and cigarette smoke. Autophagy, a cellular degradation pathway directs cells to maintain their homeostatic function such as protein turnover. Autophagy is a cellular adaptive defense mechanism and occurs in stressful environments such as during low nutrient situations, high energy demands, infection, following organelle damage, etc. In general, the autophagic process involves formation of autophagosome that fuses with lysosome resulting in formation of autolysosome that is responsible for breakdown and recycling of sequestered cytosolic material. In mammalian cells, the conversion of LC3B protein from its free form, LC3B-I to the phosphatidylethanolamine-conjugated form, LC3B-II, is an important hallmark in monitoring the autophagic flux [16,17]. mTOR is one of the primary regulators of autophagy, which activates in nutrient rich environments, thereby inhibiting autophagy but inhibited mTOR activates the autophagic process. Different forms of illness such as cancer, neurodegenerative disorders, cardiovascular diseases, diabetes are associated with dysregulated mTOR signaling due to imbalance of nutrient and energy demands, therefore, suggesting malfunctioned autophagy [18–23]. Further, many inhalation exposures result in the dysregulation of mTOR signaling and may contribute to disease via autophagy.

### Respiratory implications

Exhaust fumes from automobiles, emissions from industries and refineries including power generating stations are major sources of outdoor pollution. In contrast, cooking, burning fuel, dry cleaning, and domestic heating activities contribute as indoor sources of pollution. The mixture of particulate matter (PM) and gases are inhaled and penetrated into lungs, leading to pulmonary and systemic health effects [24]. In healthy lung tissue, mTORC1 is basally activated and the autophagy-related protein LC3 II is suppressed, maintaining cell homeostasis, and protecting cell damage from stress. However, lung injury following exposures can result in the inhibition of mTORC1 causing the upregulation of LC3 II, leading to development of pulmonary disease. Intratracheal instillation of PM with a diameter of less than 2.5 microns (PM_2.5_) in mice lungs induces pulmonary damage with vascular hyperpermeability and inflammatory responses. Mechanistically, PM_2.5_ upregulates Toll-like receptor (TLR) expression, releasing inflammatory cytokines. Additionally, PM_2.5_ inhibits mTOR signaling pathway, decreasing the levels of phosphorylated mTOR, Protein kinase B (Akt), and PI3K [25], worsening the lung injury. Consequently, the study reported a strong connection of complex signaling network between TLR, one of the sensors for autophagy and mTOR, also involved in lung inflammation [25, 26]. These findings suggest disruption of mTOR signaling contributes to PM-induced lung damage.

The fundamental insights of risk factors such as lack of physical activity, high calorie intake and high glycemic index have been well known for decades. However, a strong link between environmental factors in air and predisposition of diabetes mellitus has been growing recently. Moreover, extensive data from high fat diet induced murine model and humans suggests ambient air pollution with PM_2.5_ exposure is critically associated with pathophysiological disturbances in metabolic disease conditions [27]. Autophagy serves as a protective mechanism in pancreatic B cell survival. Chronic activation of mTORC1 inhibits the autophagy and facilitates the progression of type 2 diabetes mellitus and insulin resistance [28–30]. Activated mTOR stimulates S6K1 to inhibit insulin receptor substrate-1 (IRS1), affecting the insulin pathway, causing insulin resistance [31–33]. mTOR also mediates lipogenesis and formation of adipose tissue. Role of S6K1 has been investigated in diet-induced obesity mice model, which suggested mice lacking S6K1 are protected against obesity [34]. In addition, the study suggests exposure to PM_2.5_ exacerbates a key defect in insulin signaling via alterations in PI3K/AKT pathway increasing inflammation at adipose tissue macrophages [27]. Prospectively, abnormalities in PI3K/AKT pathway can be suggestive of cascade activation of mTOR implicating its potential in exaggerating inflammatory response in metabolic disease.

Metal nanoparticles have a wide range of applications from medicine to consumer products and incorporation into industrial processes. With potential manufacturing and widespread use of nanotechnology, exposure to engineered nanoparticles has also increased among consumers and manufacturers. Exposure to nanoparticles composed of metals and metal oxides have potential for adverse environmental and health effects [35]. Inhalation is the primary route of nanoparticle exposure in environmental and occupational settings, resulting in pulmonary damage leading to lung injury and development of respiratory diseases [36]. Several studies have reported autophagy is a consequence of metal nanoparticle exposure contributing to toxicity [37–41]. In particular, mesoporous silica nanoparticles induce autophagy in human bronchial epithelial cells (BEAS-2B) via mTOR inhibition, in a dose-dependent manner [42]. Specifically, mesoporous silica nanoparticle exposure resulted in decreased phosphorylation of mTOR and p62 levels, suggesting induction of autophagy, which was further confirmed in mouse lungs exposed to mesoporous silica nanoparticles via intratracheal instillation [42]. This silica-induced autophagy leads to macrophage activation, stimulating lung fibroblasts activity and progression of silicosis (pulmonary fibrosis) [43]. Overall, nanoparticle inhalation exposure may cause dysregulation of mTOR signaling inducing autophagy, potentially contributing to pulmonary damage and other respiratory health concerns.

### Neurological implications

Air pollution and nanoparticle exposures have the potential to induce neurotoxicity via direct interactions following transport from the lung to the brain or indirectly via secondary mechanisms. In particular, nanomaterials have applications in medicine as drug delivery systems due to their ability to increase drug bioavailability. However, some nanomaterials have the ability to produce reactive oxygen species (ROS), inducing oxidative stress leading to detrimental effects on the brain. ROS are produced in response to xenobiotics and environmental stressors and as a by-product of oxygen metabolism. An imbalance in production and removal of these ROS by biological system is termed as oxidative stress. This highly reactive oxygen containing species induces mitochondrial dysfunction and stimulates various signaling proteins kinases. These oxidative damage conditions can initiate autophagosome formation inducing autophagy [44, 45]. In biological system, NPs tends to form protein-NP corona depending on protein affinity and NPs characteristics. Additionally, NPs functions as molecular chaperon to carry out protein folding, stabilization and protein aggregates. However, alterations in formation of stable corona and high protein concentration can result in accumulation of protein aggregates [46]. Autophagy is crucial in clearing toxic intracellular proteins and aggregates [amyloid-β (Aβ), mutant huntingtin, α-synuclein, and, phosphor-tau] which contribute to neurodegenerative diseases [47, 48]. Glycogen synthase kinase-3 beta (GSK3β), the main kinase that phosphorylates tau protein, is a component of the PI3K/AKT/mTOR signaling pathway, Increased expression of GSK3β can lead to hyperphosphorylation of tau protein via activation of mTOR, resulting in its aggregation and the formation of neurofibrillary tangles, which are a major neuropathological feature of Alzheimer’s disease [49–52]. Furthermore, it has been evidently reported that air pollution with PM_0.1_ triggers the development of central nervous system disorders such as Alzheimer’s, Parkinson’s and stroke. An investigation on exposure of PM_0.1_ composed of aluminum emphasized the generation of ROS with association of autophagosome and oxidative DNA damage in neuronal cells. Interestingly, nanoparticles are proposed to cause autophagy by oxidative stress mechanism resulting in mitochondrial damage and accumulation of proteins. Hence, the likelihood of PM_0.1_ exposure generating ROS that can induce autophagy contributing to neuronal damage and development of neurodegenerative diseases should not be disregarded [46].

Modern general anesthesia methods ensure the safety of numerous surgeries each year [53]. In clinical settings, the most utilized inhalational anesthetics are isoflurane, sevoflurane, and desflurane. Research on the safety of these anesthetics has indicated they can have both beneficial and harmful impacts on various organs, including the lungs, trachea, diaphragm muscle, liver, and kidneys [54]. Further, the use of anesthetic agents could potentially cause harm to the development of neurocognition, particularly during early childhood. Sevoflurane is a widely and popularly used inhalation anesthetic in children. It has been proposed that infant exposure to sevoflurane and isoflurane are associated with long-term loss of cognitive functions and neurobehavioral changes [55–57]. Autophagy is crucial for normal brain development since it is involved in nutritional supplementation during the prenatal period [58, 59]. Interfering with autophagy can disturb the proper coordination of cell growth, specialization, and programmed cell death that are required for developing the intricate structure of the nervous system [59]. This dysregulated autophagy was substantially studied in pregnant rats exposed to sevoflurane. It was determined inhalation of sevoflurane upregulated autophagy via the PTEN/Akt/mTOR pathway in fetus brains [58]. Further, phosphatase and tensin homolog on chromosome 10 (PTEN), is abundantly expressed in embryonic central nervous system and affects brain development. In mammalian cells, PTEN can modulate autophagy by counteracting PI3K via its lipid phosphatase function. Specifically, compared to the control group, the administration of sevoflurane resulted in a noteworthy increase of PTEN expression and a decrease in the levels of p-Akt/Akt and mTOR in fetal brains. This suggests the combined involvement of PTEN/Akt/mTOR signaling in activating autophagy in sevoflurane-induced neurotoxicity [58]. Broadly, these results suggest systemic effects following inhalation exposures could be mediated by variations in mTOR signaling.

## Inhalation exposure modified mTOR signaling contributing to disease via lipid Metabolism and inflammation

mTOR, a primary regulator of cellular growth and development, also has key translational and/or transcriptional roles in inflammation with implications in atherosclerosis and other inflammatory diseases [60]. Pulmonary epithelial cells are primary target for exogenous irritants and act as a first line defense to protect against the inhaled particulates of air including pollutants, pathogens, allergens, etc. When exposed to foreign substances these epithelial cells not only restrict the systemic entry but also can activate the immune response [61]. mTOR activity is an important intracellular regulator of leptin-induced macrophage lipid metabolism. Leptin, a hormone/cytokine secreted by adipocytes, modulates appetite control, and regulates energy metabolism via interactions with the hypothalamus. Interestingly leptin is directly involved in the formation of lipid droplets via activation of epithelial cells. Leptin directly exaggerates the production of inflammatory mediators such as leukotriene B4 via activation of the mTOR pathway. Specifically, in macrophages, leptin activates PI3K/mTOR pathway, resulting in formation of lipid droplets, which are otherwise inhibited by rapamycin treatment (mTORC1 inhibitor). Lipid droplets are sites where lipid metabolism enzymes such prostaglandin-endoperoxide synthase 2 (PTGS2, also known as cyclooxygenase 2 or COX-2) involved in production of prostaglandins are located. Hence the formation of lipid droplets demonstrated increased expression of COX-2 and phospholipase A_2_ protein expression suggesting of pro-inflammatory response [62]. Moreover, exposure to PM_2.5_ increases the adipose tissue macrophages which parallelly increases the visceral adipose tissue content and size, secreting more proinflammatory substances, causing inflammation [63]. Mechanistically, the findings confirm that increases in visceral fat causes dysregulation in PI3K/Akt/endothelial NO synthase signaling, causing inflammation and insulin resistance [27]. Overall, one could conclude, mTOR mediates metabolic syndrome and obesity-induced inflammation that is exaggerated by inhalation exposure.

### Pulmonary outcomes

It is widely acknowledged that cigarette smoking is the primary contributor to the development of lung cancer due to the strong correlation between the quantity of cigarettes consumed and the likelihood of developing the disease. The main cause of COPD is the inhalation of chemicals and oxidants present in cigarette smoke (CS), in addition to being exposed to dust, industrial, or household particles [64, 65]. COPD is marked by a cumulative and complete blockage of the airways (known as chronic bronchitis), damage to the alveolar walls (emphysema), and excess mucus production [66]. Cysteine cathepsins are important in lung homeostasis and their dysregulation plays a key role in lung injury and inflammation via degrading antimicrobial peptides and extracellular matrix components. Murine experimental emphysema reported to have increased levels of cathepsin S, which is a potent elastase involved in alveolar remodeling [67–69]. Specifically, nicotine-containing cigarette smoke extract (CSE) upregulates cathepsin expression in human macrophages via mTOR pathway, modulating epithelial integrity and enhancing its permeability in COPD. This was confirmed when macrophages exposed to nicotine-containing CSE and nicotine alone demonstrated similar fold increase in cathepsin upregulation suggesting nicotine to be driving factor of responses [64]. Furthermore, the signaling pathway was confirmed with treatment of rapamycin, a known mTOR inhibitor, which demonstrated increase in immunoreactive and mature cathepsin and decrease in phosphorylated transcription factor EB (TFEB), a central regulator of autophagy/lysosomal biogenesis [70]. Consequently, dysregulation of mTOR signaling contributes to CS-associated induction of pulmonary inflammation and disease.

### Cancer

Prevalently, 90% of lungs cancer diagnosis is associated with the use of tobacco. Tobacco smoke contains numerous components including aromatic amines and polychromatic hydrocarbons that promote carcinogenesis. Additionally, these components, especially nicotine and nitrosamine 4-(methylnitrosamino)-1-(3-pyridyl)-1-butanone (NNK) produce active metabolites that promote DNA adduct formation. The carcinogenic effects of tobacco components involve activation of multiple signaling pathways that regulate cell cycle and proliferation. Of them, Akt/mTOR pathway is critically involved cell growth, metabolism, and survival. Abnormal and excessive mTOR activation promotes tumor growth, metastasis, and angiogenesis, a process that supplies carcinoma cells with oxygen and nutrients. Particularly, tobacco components damages DNA by activating Akt pathway and modulates PI3K, upstream regulator of Akt [71]. Experimentally, immunohistochemistry and immunoblotting on lung tissues of NNK-treated A/J mice reveals presence of phosphorylated Akt in airway epithelial cells and 5.5-fold increase in ratio of phosphorylated Akt to total Akt compared to phosphate buffered saline (PBS) control mice, respectively [72]. Similarly, *in vitro* studies on nicotine or NNK treated non-small cell lung cancer cells reports to increase phosphorylation of S6K1 and 4E-BP1, activate mTOR and induce cell proliferation [72, 73]. In short, tobacco smoke components foster cell proliferation and tumorigenesis via mTOR activation.

## Conclusion

Inhalation exposure induces pulmonary and systemic effects contributing to the pathogenesis of various diseases. Inhaled pollutants such as metal nanoparticles, exhaust fumes, cigarette smoke, and some anesthetics induce several detrimental cellular processes causing pathological outcomes. mTOR is involved in numerous cellular processes including cellular growth and metabolism. Given its importance in homeostasis, the dysregulation in mTOR signaling contributes to a variety of disease outcomes. mTOR is primarily regulated by PI3K/AKT signaling which can directly and/or indirectly be modified by many inhalation exposures resulting in autophagy, altered lipid metabolism, and inflammation. These cellular processes are known to contribute to multiple pulmonary and systemic diseases. Treatments focusing on reestablishing regulation of mTOR signaling could potentially be helpful in addressing inhalation exposure-induced diseases.
